# Drug Resistance Patterns of* Escherichia coli* in Ethiopia: A Meta-Analysis

**DOI:** 10.1155/2018/4536905

**Published:** 2018-05-06

**Authors:** Kald Beshir Tuem, Abadi Kahsu Gebre, Tesfay Mehari Atey, Helen Bitew, Ebrahim M. Yimer, Derbew Fikadu Berhe

**Affiliations:** ^1^Department of Pharmacology and Toxicology, School of Pharmacy, College of Health Sciences, Mekelle University, Mekelle, Ethiopia; ^2^Clinical Pharmacy Unit, School of Pharmacy, College of Health Sciences, Mekelle University, Mekelle, Ethiopia; ^3^Department of Pharmacognosy, School of Pharmacy, College of Health Sciences, Mekelle University, Mekelle, Ethiopia

## Abstract

**Background:**

Antimicrobial drug resistance is a global threat for treatment of infectious diseases and costs life and money and threatens health delivery system's effectiveness. The resistance of* E. coli* to frequently utilized antimicrobial drugs is becoming a major challenge in Ethiopia. However, there is no inclusive countrywide study. Therefore, this study intended to assess the prevalence of* E. coli* resistance and antimicrobial-specific resistance pattern among* E. coli* clinical isolates in Ethiopia.

**Methods:**

Articles were retrieved from PubMed, Embase, and grey literature from 2007 to 2017. The main outcome measures were overall* E. coli* and drug-specific resistance patterns. A random-effects model was used to determine pooled prevalence with 95% confidence interval (CI), using DerSimonian and Laird method. In addition, subgroup analysis was conducted to improve the outcome. The study bias was assessed by Begg's funnel plot. This study was registered in PROSPERO as follows: PROSPERO 2017: CRD42017070106.

**Results:**

Of 164 articles retrieved, 35 articles were included. A total of 19,235 study samples participated in the studies and 2,635* E. coli* strains were isolated. Overall,* E. coli* antibacterial resistance was 45.38% (95% confidence interval (CI): 33.50 to 57.27). The resistance pattern ranges from 62.55% in Addis Ababa to 27.51% in Tigray region. The highest resistance of* E. coli* reported was to ampicillin (83.81%) and amoxicillin (75.79%), whereas only 13.55% of* E. coli* isolates showed resistance to nitrofurantoin.

**Conclusion:**

* E. coli* antimicrobial resistance remains high with disparities observed among regions. The bacterium was found to be highly resistant to aminopenicillins. The finding implies the need for effective prevention strategies for the* E. coli* drug resistance and calls for multifaceted approaches with full involvement of all stakeholders.

## 1. Background

*Escherichia coli (E. coli) *is one of the most widespread bacteria throughout the world. Some strains of* E. coli* can cause serious illness for humankind [1] including urinary tract infections [2–4], bloodstream infections [5], skin infection, otitis media [6, 7], and diarrhea [8].

*E. coli* resistance to antimicrobials is creating trouble to the healthcare system worldwide [9, 10]. This complicates treatment outcomes, increases the cost of treatment, and limits the therapeutic options that contribute to the global spectra of a postantimicrobial age in which some of the most effective drugs lose their efficiency [11]. The bacterium is becoming highly resistant to conventionally used antibiotics (to both the newer and older medicines) as evidenced by many previous studies [12–16]. Adaptive resistance was supposed to be the main mechanism for the development of resistance including that to lethal doses of the antimicrobials [17].

Antimicrobial resistance of* E. coli* in developing countries including Ethiopia is reported to be one major reason for failure of treatment of infectious diseases [18]. A number of studies conducted in Ethiopia from various clinical settings show increments in the prevalence of antimicrobial resistance patterns of* E. coli* [6, 19, 20]. However, there is no comprehensive and aggregated nationwide study to show the pattern of antimicrobial resistance in* E. coli*. Hence, the purpose of this meta-analysis was to sum up the available data and to establish the pooled prevalence and antimicrobial resistance of* E. coli* in Ethiopia.

## 2. Methods

This study was conducted in a similar approach to Eshetie et al. (2016) [21] and according to the Preferred Reporting Items for Systematic Reviews and Meta-Analysis (PRISMA) Checklist [22] (additional S1 file).

### 2.1. Study Selection

A systematic literature search was conducted in PubMed and Embase, and also manual search for articles potentially relevant to our study was identified. We built our search strategy by combining the three main arms (Table 1):* E. coli*, drugs-related terms, and Ethiopia.

Among the citations extracted, abstracts were reviewed to retrieve the clinical studies on* E. coli* colonization. Articles that were relevant, by title and abstract, were accessed in full text to determine those that provided sufficient information to be included in our meta-analysis. Finally, the references cited by each eligible study were screened to identify additional articles.

### 2.2. Inclusion and Exclusion Criteria

Studies included in this meta-analysis were those that had extractable data on the prevalence of drug resistance of* E. coli* on a human in Ethiopian hospitals or research centers and were only published from 2007 to 2017 and were only in English language.

### 2.3. Outcome of Interest

The main outcome of interest was the prevalence of drug resistance or antimicrobial susceptibility of* E. coli* among the total* E. coli* clinical isolates. The prevalence was calculated by dividing the numbers of resistant* E. coli* isolates by the total number of clinically isolated* E. coli*. As a secondary outcome of interest, we had also calculated the pooled resistance pattern of* E. coli* isolates to specific antibiotics.

### 2.4. Data Extraction and Assessment of Quality of Study 

Screening by title, abstract, and full text and data extraction were done independently by two authors (Kald Beshir Tuem and Abadi Kahsu Gebre) at each step and Derbew Fikadu Berhe was involved in consensus for discrepancies (if any) between the two authors (Kald Beshir Tuem and Abadi Kahsu Gebre). In cases of insufficient data, the authors reviewed the full text of the article for further information and clarification. The extracted data from each article were summarized into a spreadsheet. References and data for each study were carefully cross-checked to ensure that no overlapping data were present and to maintain the integrity of the meta-analysis. Information extracted from each paper was region, study area, study design, study population, culture specimens, number of* E. coli* isolated, the average percentage of resistant* E. coli*, antimicrobial resistance rate of* E. coli*, and references. With all the articles used in this study being cross-sectional, the score for the quality of the study was assessed using the modified Newcastle-Ottawa scale for the representativeness of sample, appropriateness of sample size, response rate, validity of method, strategy to control confounding factors, reliability of outcome determination, and appropriate statistical analyses. The quality score (Table 2) disagreements were resolved by consensus and a final agreed-upon rating was assigned to each study (S2 file) [58].

### 2.5. Quality Control

The quality of eligible studies was checked independently by two authors (Kald Beshir Tuem and Abadi Kahsu Gebre) using a set of predetermined criteria such as research design quality of paper, completeness of extractable information, and employed methods for* E. coli* isolation. The study bias was measured by Begg's funnel plot [59]. This study was registered in PROSPERO as follows: PROSPERO 2017: CRD42017070106.

### 2.6. Data Analysis

A random-effects model was used to determine pooled prevalence, subgroup analysis, and 95% confidence interval (CI) by employing the approach of DerSimonian and Laird [60]. Variances and CIs were stabilized using Freeman-Tukey arc-sine methodology [61]; the reason is that using the standard approach of inverse variance method to calculate pooled prevalence does not work well in meta-analysis of single-arm study because, for studies with small or large prevalence, the inverse variance method causes the variance to become small and the calculated CI may be outside of the range [62]. Heterogeneity of study results was assessed using *I*^2^ test and significant heterogeneity was considered at *p* < 0.10 and *I*^2^ > 50% [60, 63]. Statistical analyses were performed using Open Meta-Analyst (version 3.13) and Comprehensive Meta-Analysis (version 3.1). In addition, we performed subgroup analyses according to the region of the country and the mechanism of action of the tested drugs to improve the specificity of the assessment.

## 3. Results

**Figure 1:**
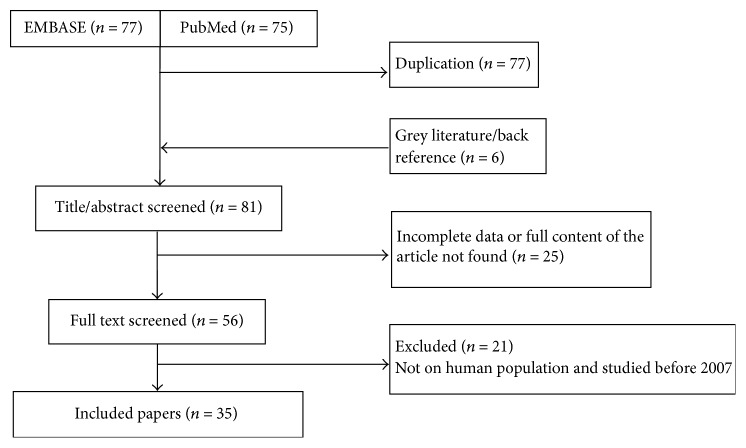
Flowchart shows selected articles for meta-analysis.

From Embase, PubMed, and manual searching, we found 164 potentially relevant studies, of which 35 were included for analysis (Figure 1).

**Figure 2:**
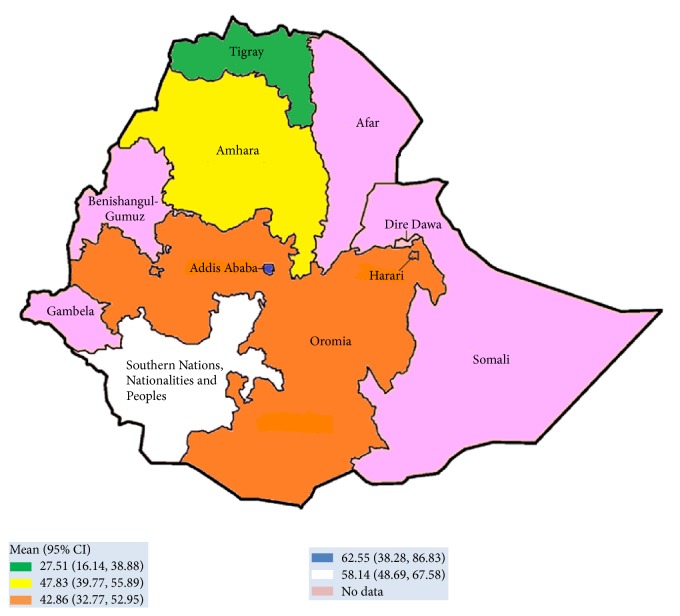
Proportion of* E. coli* resistance in different regions of Ethiopia, 2007–2017. Values in parenthesis indicated 95% CI of* E. coli* resistance in different regions of Ethiopia.

**Figure 3:**
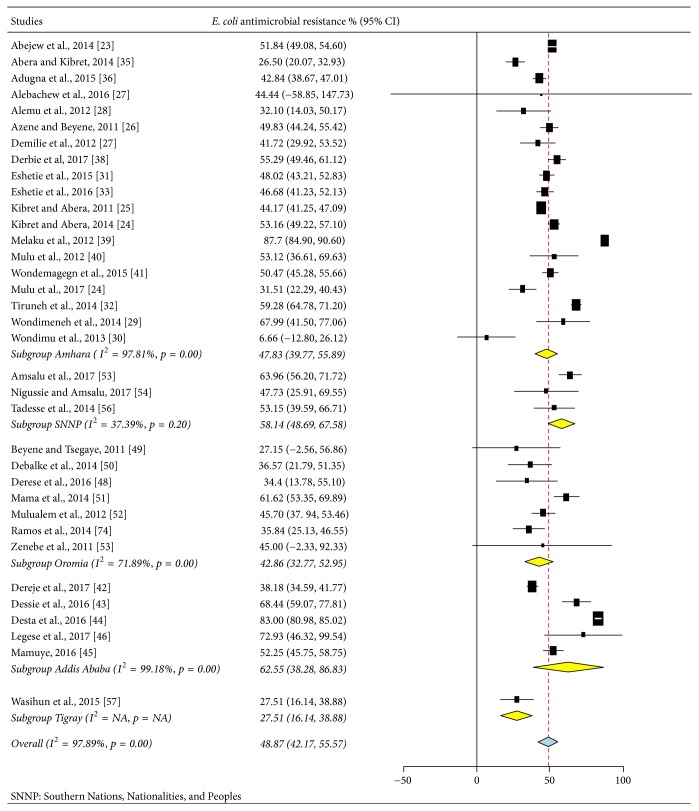
Subgroup analysis of* E. coli* antibacterial resistance according to regions of Ethiopia.

The study design of all included articles (35) was cross-sectional study. In these studies, a total of 19,235 study samples participated, from which 2,635* E. coli* strains were isolated. The published studies were from four regions in Ethiopia (Table 2) which include the federal capital city of Ethiopia, Addis Ababa. No report was obtained from other regions in the country (Afar, Benishangul-Gumuz, Gambella, and Somali). Most of the studies indicated that various specimens had been utilized for screening of* E. coli*; particularly multisite swabbing was performed from different parts of the body, including skin, nasal, eye, ear, urethra, throat, vagina, or genital area (Table 2), and other biological fluids like blood, urine, pus, stool, and cerebrospinal fluid (CSF) were taken for test. A total of 2,635* E. coli* strains were isolated from these various sites. The lowest and highest proportions of* E. coli* resistance were reported, respectively, from Bahir Dar (55.20%) and Mekelle (27.50%) cities. The average prevalence of* E. coli* resistance was also noted in different regions of Ethiopia; Addis Ababa region was ranked first (62.55%, 95% CI: 38.28–6.83%), followed by Southern Nations, Nationalities, and Peoples of Ethiopia (58.14%, 95% CI: 48.69–67.58%), Amhara (47.83%, 95% CI: 39.77–55.89%), and Oromia (42.86%, 95% CI: 32.77–52.95%), whereas relatively low magnitude of* E. coli* resistance was reported from Tigray region (27.51%, 95% CI: 16.14–38.88%) (Figures 2 and 3).

Subgroup analyses (Figure 3) were carried out based on the region (Addis Ababa, Amhara, Oromia, SNNP, and Tigray) and the mechanism of action of the drugs (cell wall synthesis inhibitors, protein synthesis inhibitors, DNA synthesis inhibitors, and antimetabolites). A paper-based analysis in our study showed that the overall* E. coli* resistance in Ethiopia was 48.87% (95% CI: 42.17–55.57%) with highest prevalence in the capital city, Addis Ababa, 62.55% (95% CI: 38.28–86.83%) (Figure 3).

**Figure 4:**
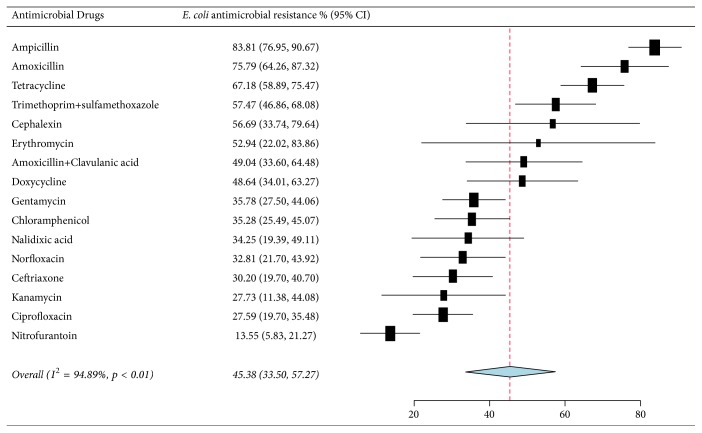
Forest plot of the pooled percentage and confidence interval of* E. coli* resistance to antibacterial drugs in Ethiopia from 2007 to 2017.

As presented in Figure 4 and Table 3, the pooled prevalence of* E. coli* resistance was 45.38% (95% CI: 33.50–57.27%), and high resistance rates were observed to ampicillin, 83.81% (95% CI: 76.95–90.67%), amoxicillin, 75.79% (95% CI: 64.26–87.32%), tetracycline, 67.18% (95% CI: 58.89–75.47%), trimethoprim-sulfamethoxazole, 57.47% (95% CI: 58.89–75.47%), and cephalothin, 56.69% (95% CI: 33.74–79.64%). A relatively low level of nitrofurantoin resistance was observed, 13.55% (95% CI: 5.83–21.27%).

**Figure 5:**
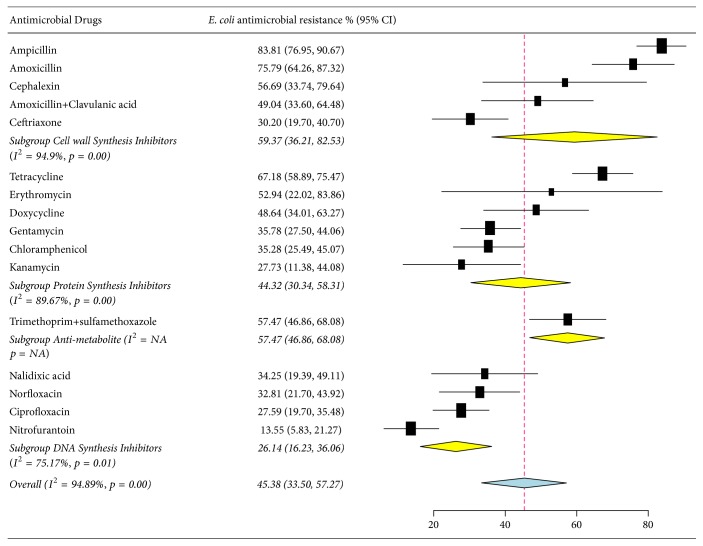
Subgroup analysis of pooled percentage and confidence interval of* E. coli* resistance to antibacterial drugs according to drug mechanism of action.

Comparing the prevalence of* E. coli* resistance among the antibacterial drugs, subgroup analysis (Figure 5) revealed that the cell wall synthesis inhibitors account for the greatest resistance percentage, 59.37% (95% CI: 36.21–82.53%), and DNA synthesis inhibitors account for the lowest resistance percentage, 26.14% (95% CI: 33.50–57.27%).

There was a high level of heterogeneity by random model methods (*I*^2^ = 97.89%; *p* < 0.01). Hence, the included studies have been conducted in different study settings, study periods, and study populations, which could have an effect on the heterogeneity of the included studies. The symmetry of funnel plot showed small study bias, which yielded insignificant effect.

## 4. Discussion

Antibiotic resistance continues to be a major global challenge in the management of bacterial infection. The trouble behind antibiotic resistance is highly marked in undeveloped or developing countries, including Ethiopia, where infectious diseases are highly prevalent [64]. Factors responsible for an increase in rates of antimicrobial resistance include misuse/overuse of antibiotics by healthcare professionals and general public and inadequate surveillance systems due to lack of reliable microbiological techniques leading to the inappropriate prescription of antibiotics [33]. Antimicrobial resistance in* E. coli* has increased worldwide and its susceptibility patterns show substantial variation in different geographical locations [5]. To date, the overall epidemiology and burden of multidrug resistance (MDR) bacteria have not been fully understood, especially in resource-limited countries including Ethiopia [64, 65]. To the best of our knowledge, this is the first meta-analysis study conducted to determine the pooled prevalence of* E. coli* prevalence and resistance in Ethiopia. Our result revealed that* E. coli* strains displayed diverse resistance patterns, with percentages varying slightly based on sample type and geographical distribution. Based on the tested antimicrobials, the overall* E. coli* resistance in Ethiopia was nearly 50% (45.38% (95% CI: 33.50–57.27%)).

Developing countries have comparatively higher risk factors associated with MDR strains than the developed ones [64, 66]. Resistance to antibacterial agents is a normal evolutionary process for microorganisms, but it is highly aggravated by continuous deployment of antimicrobial drugs in treating infections [67, 68]. It is claimed that more than half of drugs are prescribed, sold, or dispensed without following standard protocols, and the situation is more pronounced in developing countries including Ethiopia [69]. Sosa et al. (2010) reported that antibiotic usage in most of the low-income countries is generally unregulated, which is a prime factor for the occurrence of resistant bacterial strains [64]. This implies that antibiotics are being used widely and inappropriately in resource-limited countries including Ethiopia. This may lead to an increase in the occurrence of drug-resistive bacterial strains such as* E. coli*.

In our study, the regional prevalence of* E. coli* resistance was estimated, and the subgroup analysis showed that the highest prevalence of* E. coli* resistance (62.55%) was noted in Addis Ababa city, which was almost two times higher than Tigray region (27.51%). The observed variation might be due to differences in study location, hospital setup, and antimicrobial utilization.

Subgroup analysis also showed that* E. coli* strains exhibited higher resistance with cell wall inhibitors, specifically aminopenicillins (ampicillin and amoxicillin), followed by protein synthesis inhibitors, mainly to tetracycline, and lesser resistance prevalence to nitrofurantoin. In line with our data, globally,* E. coli* strains were reported to be highly resistant to the above-mentioned antibiotics, mainly to aminopenicillins [70, 71]. Săndulescu (2016) reported that* E. coli* showed low resistance to nitrofurantoin, which is in line with the present finding.

Moreover, our finding indicated the higher magnitude of* E. coli* resistance. This may imply the need for intervention in prescribing and using antibacterial against* E. coli* infections. Interventional strategies may include creating public awareness, maintaining hand hygiene, applying infection prevention protocols, and maintaining environmental sanitation, which are encouraged for preventing infection. In addition to these, promoting health education, maintaining continuous professional educations, and advocating rational prescribing habits are evidently effective in the minimization of the unwanted use of antibiotics, which in turn decrease selective pressure of resistant strains.

## 5. Conclusion

In this meta-analysis, the pooled* E. coli* resistance is considerably high.* E. coli* strains were highly resistant to ampicillin but showed lesser resistance to nitrofurantoin. Adopting safety protocols and implementing proper antibiotic prescription policies could be potential interventional strategies to address the emerging resistance of* E. coli*.

## Figures and Tables

**Table 1:** Searching strategies including search arm and terms used in the study.

	Search arm	Search terms
1	*E. coli*	“*Escherichia coli*” OR *“E. coli”*
2	Drug and/or resistance	“antibiotic resistance” OR “drug resistance” OR “drug resistance, microbial” OR “drug resistance, microbial” OR “antibacterial resistance” OR “antibiotic resistance” OR “antimicrobial resistance”
3	Ethiopia	“Ethiopia”

The word “OR” was used to combine search terms within each arm and the word “AND” was used to connect the three search arms (#1 AND #2 AND #3).

**Table 2:** Summary of 35 studies reporting the prevalence and resistance pattern of *E. coli *in different parts of Ethiopia.

References	Region	Study area	Study period	Study design	Study population	Culture specimens	Study samples	Number of *E. coli* isolated	Average %resistant *E. coli*	NOS quality score out of 7
Abejew et al., 2014 [23]	Amhara	Dessie	January to March 2012	RCS	Test results of patients diagnosed with UTI	Urine	2486	410	51.8	6
Kibret and Abera, 2014 [24]		Dessie	—	R	Patients	Mid-stream morning urine	1404	203	53.2	6
Kibret and Abera, 2011 [25]		Dessie	—	R	Patients	Urine, ear discharge, pus swab from wounds, and eye discharge	3,149	446	44.2	6
Azene and Beyene, 2011 [26]		Dessie	—	R	Patents with wound infections	Wound swab	599	82	49.8	6
Alebachew et al., 2016 [27]		Gondar	March 1 to May 2, 2013	CS	HIV patients	Blood	100	01	44.4	7
Alemu et al., 2012 [28]		Gondar	March 22 to April 30, 2011	CS	Pregnant women attending ANC	Urine	385	19	29.2	7
Wondimeneh et al., 2014 [29]		Gondar	January to May 2013	CS	Fistula patients	“Clean-catch” mid-stream urine specimen	53	6	59.3	6
Wondimu et al., 2013 [30]		Gondar	December 2011 to June 2012	CS	Blood donated from donors at blood bank	blood	137	2	0.7	7
Eshetie et al., 2015 [31]		Gondar	February to May 2014	CS	UTI-suspected patients	Urine	442	112	48.0	4
Tiruneh et al., 2014 [32]		Gondar	September 1, 2011, to June 30, 2012	CS	UTI-suspected patients	Urine	284	120	68.0	4
Eshetie et al., 2016 [33]		Gondar	February to June 2014	CS	Patients with UTI	Urine	446	112	38.8	6
Mulu et al., 2017 [34]		Debre Markos	January 2015	R	Patients	Pus/swab from wound, urine, ear discharge, blood, stool, urethral or cervical discharge, nasal or throat swab, and CSF	575	39	31.5	6
Abera and Kibret, 2014 [35]		West Gojjam	November 2009 to February 2010	CS	Adult patients who underwent trachomatous trichiasis surgery	Conjunctival swabs	1413	20	26.5	5
Adugna et al., 2015 [36]		Bahir Dar	December 2011 to February 2012	CS	Children below five years of age with acute diarrhea	Stool sample	422	204	42.8	7
Demilie et al., 2012 [37]		Bahir Dar	October 2010 to January 2011	CS	Pregnant women	Clean-catch urine	367	16	41.7	4
Derbie et al., 2017 [38]		Bahir Dar	January 2015	R	Patients	Urine	446	72	55.3	5
Melaku et al., 2012 [39]		Bahir Dar	April to August 2010	CS	Patients	Urine	1254	33	87.8	6
Mulu et al., 2012 [40]		Bahir Dar	October 2010 to January 2011	CS	Patients	Wound swab and venous blood samples	294	8	53.1	6
Wondemagegn et al., 2015 [41]		Bahir Dar	May to November 2013	CS	Women of reproductive age	Vaginal swab	409	105	50.5	7

Dereje et al., 2017 [42]	Central Ethiopia	Addis Ababa	February to May 2015	CS	Fistula patients	Clean-catch mid-stream urine	210	65	38.2	7
Dessie et al., 2016 [43]		Addis Ababa	October 2013 and March 2014	CS	Surgical site infected patients	Wound swab	107	24	68.4	6
Desta et al., 2016 [44]		Addis Ababa	December 2012	CS	All age groups patients	Fecal samples/swabs	267	235	83.0	7
Mamuye, 2016 [45]		Addis Ababa	August 2013 to January 2014	CS	Outpatient and inpatient and pregnant women	Mid-urine samples	424	53	52.2	5
Legese et al., 2017 [46]		Addis Ababa	January to March 2014	CS	Septicemia and UTI-suspected patients	Blood and urine	322	6	72.9	6

Ramos et al., 2014 [47]	Oromia	West Arsi	July to December 2013	CS	Leprosy patients	Pus produced by ulcer	68	17	35.8	5
Derese et al., 2016 [48]		Dire Dawa	February 18, 2015, to March 25, 2015	CS	Pregnant women	Urine specimens	186	9	34.4	6
Beyene and Tsegaye, 2011 [49]		Jimma	April to June 2010	CS	UTI cases patients	Urine	228	7	27.1	5
Debalke et al., 2014 [50]		Jimma	September to December 2012	CS	HIV/AIDS patients	Urine	481	31	36.6	7
Mama et al., 2014 [51]		Jimma	May to September 2013	CS	Patients with wound infection	Wound swab	150	29	61.6	6
Mulualem, 2012 [52]		Jimma	February to March 2007	CS	Inpatients and outpatients	Urine, sputum, stool, and wound	359	67	45.7	6
Zenebe et al., 2011 [53]		Jimma	October 27, 2009, to March 26, 2010	CS	Febrile patients	Venous blood	260	4	25.0	6

Nigussie and Amsalu, 2017 [54]	SNNP	Hawassa	June to October 2014	CS	Diabetic patients	Mid-stream urine	240	11	47.7	7
Amsalu et al., 2016 [55]		Hawassa	January 2012 to December 2014	RCS	Patients registered at microbiology lab book	Pus, ear discharge, nasal swab, urine, genital swab, CSF, and stool	510	35	64.0	7
Tadesse et al., 2014 [56]		Hawassa	March to September 2012	CS	Pregnant women attending ANC	Mid-stream urine	244	16	56.3	6

Wasihun et al., 2015 [57]	Tigray	Mekelle	March to October 2014	PCS	Febrile patients	Venous blood	514	16	27.5	6

					*Total*		*19,235*	*2,635*		

ANC: antenatal clinic, CSF: cerebrospinal fluid, CS: cross-sectional, PCS: prospective cross-sectional, R: retrospective review of culture, RCS: retrospective cross-sectional, SNNP: Southern Nations, Nationalities, and Peoples, UTI: urinary tract infection, —: no data.

**Table 3:** Percentage of pooled antibacterial resistance rates of *E. coli* in Ethiopia, 2007–2017.

Studies	Antibacterial															
	AML	AMP	AMC	KF	CRO	TE	DO	C	E	CN	K	CIP	NA	F	NOR	SXT
Abejew et al., 2014 [23]	84.6	80.0	---	42.3	46.9	82.2	64.5	49.0	94.4	34.0	20.0	28.3	13.3	10.4	---	75.8
Abera and Kibret, 2014 [35]	---	---	---	---	40	25	---	35	---	40	---	4	---	---	---	15
Adugna et al., 2015 [36]	---	86.8	47.5	---	---	76	---	36.2	---	37.2	---	6.9	---	---	9.3	---
Alebachew et al., 2016 [27]	---	100	100	---	0	100	---	0	---	0	---	0	---	0	---	100
Alemu et al., 2012 [28]	100	100	36.8	---	0	52.6	---	0	---	5.3	---	0	---	---	0	26.3
Beyene and Tsegaye, 2011 [49]	100	100	---	---	0	28.6	---	0	---	0	---	14.3	0	0	---	28.6
Debalke et al., 2014 [50]	---	88	---	---	4	---	---	0	---	---	---	---	52	0	16	96
Demilie et al., 2012 [37]	75	81.7	37.5	---	---	68.8	---	37.5	---	31.2	18.8	18.8	43.8	6.3	25	56.2
Derbie et al., 2017 [38]	---	89.1	78.6	---	---	66.1	---	---	---	27.6	---	64.4	---	25	27	64.5
Dereje et al., 2017 [42]	---	---	21.6	---	24.6	---	---	32.2	---	53.8	---	56.9	---	40	---	---
Derese et al., 2016 [48]	77.8	77.8	---	---	0	66.7	---	11.1	---	0	---	11.1	44.4	44.4	---	11.1
Dessie et al., 2016 [43]	---	95.8	70.8	---	83.3	83.3	---	25	---	54.2	---	66.7	---	---	---	---
Desta et al., 2016 [44]	---	---	93	98	---	---	---	---	---	63	---	78	---	---	---	---
Eshetie et al., 2015 [31]	---	92.9	60.7	---	17.9	53.6	---	64.3	---	57.1	---	10.7	25	---	---	50
Kibret and Abera, 2014 [24]	87.3	---	---	56.9	33.9	80.1	61	37.4	93.7	24.5	---	34.1	---	3.8	---	72.1
Kibret and Abera, 2011 [25]	86	---	---	59.5	37.4	72.4	---	35.3	89.4	13	---	19.9	---	3.6	6.5	62.9
Azene and Beyene, 2011 [26]	85.0	---	---	64.7	66.7	71.4	33.3	36.1	51.9	14.4	---	7.7	---	---	---	67.1
Mama et al., 2014 [51]	---	100	---	100	62	79	44.8	65.5	---	51.7	---	34	41	---	44.8	55
Mamuye, 2016 [45]	75.5	79.2	---	32.1	45.3	83.0	71.7	30.2	---	22.6	---	54.7	73.6	20.8	67.9	22.6
Melaku et al., 2012 [39]	85.7	100	---	---	---	81.6	---	83.7	---	---	---	---	---	---	---	---
Mulu et al., 2017 [34]	25	33.3	---	---	3.3	75	75	13	0	69.6	---	18.2	---	---	23.1	11.1
Mulualem et al., 2012 [52]	86	86	70.1	---	9	73.1	---	35.8	---	3	---	20.9	---	---	16.4	56.7
Mulu et al., 2012 [40]	90	78	---	---	55.6	66.7	66.7	0	---	44.4	44.4	44.4	66.7	22.2	44.4	67
Nigussie and Amsalu, 2017 [54]	---	100	36.4	---	63.6	---	---	---	---	72.7	---	18.2	---	0	9.1	81.8
Ramos et al., 2014 [47]	64.7	70.6	5.9	---	5.9	41.2	29.4	35.3	41.2	11.8	---	29.4	---	---	---	58.8
Wasihun et al.2015 [57]	---	---	6.7	---	60	---	40	---	---	13.3	---	6.7	---	26.7	60	6.7
Wondimeneh et al., 2014 [29]	50	66.7	---	---	50	100	---	16.7	---	66.7	---	50	---	---	66.7	66.7
Wondimu et al., 2013 [30]	---	33.3	0	---	0	0	0	0	0	0	---	33.3	---	---	---	0
Zenebe et al., 2011 [53]	0	100	---	0	0	75	---	100	---	75	---	0	0	---	---	100
Amsalu1 et al., 2016 [54]	---	100	26.1	---	45.7	---	---	64.3	---	60.6	---	56.2	---	---	68.8	90
Tadesse et al., 2014 [56]	---	68.8	---	---	---	---	---	---	---	43.8	---	---	---	---	18.8	81.2
Tiruneh et al., 2014 [32]	87.5	83.3	---	---	---	79.2	---	58.3	---	44.2	---	46.7	---	---	---	76.7
Legese et al., 2017 [46]	100	---	100	---	---	83.3	---	66.7	---	66.7	---	---	---	0	66.7	100
Eshetie et al., 2016 [33]	---	98.2	42	---	---	43.8	---	54.5	---	52.7	---	0.9	17	---	---	64.3
Wondemagegn et al., 2015 [41]	80	73.3	---	---	---	73.3	---	---	---	26.7	---	20	---	---	20	60

AMC: amoxicillin-clavulanic acid, AML: amoxicillin, AMP: ampicillin, C: chloramphenicol, CIP: ciprofloxacin, CN: gentamicin, CRO: ceftriaxone, DO: doxycycline, E: erythromycin, F: nitrofurantoin, K: kanamycin, KF: cephalothin, NA: nalidixic acid, NOR: norfloxacin, SXT: trimethoprim/sulfamethoxazole, TE: tetracycline, ---: not done.

## Abbreviation

ANC: Antenatal clinic
AMC: Amoxicillin-clavulanic acid
AML: Amoxicillin
AMP: Ampicillin
C: Chloramphenicol
CI: Confidence interval
CIP: Ciprofloxacin
CN: Gentamicin
CRO: Ceftriaxone
CS: Cross-sectional
CSF: Cerebrospinal fluid
DO: Doxycycline
E: Erythromycin
*E. coli*: * Escherichia coli*
F: Nitrofurantoin
K: Kanamycin
KF: Cephalothin
MDR: Multidrug resistance
NA: Nalidixic acid
NOR: Norfloxacin
PCS: Prospective cross-sectional
R: Retrospective review of culture
RCS: Retrospective cross-sectional
SNNP: Southern Nations, Nationalities, and Peoples
SXT: Trimethoprim/sulfamethoxazole
TE: Tetracycline
UTI: Urinary tract infection.

## Supplementary Materials

Supplementary 1 S1 file: PRISMA 2009 Checklist. We have conducted the meta-analysis according to Moher et al. (2009), “Preferred Reporting Items for Systematic Reviews and Meta-Analyses: The PRISMA Statement.” We included all the criteria on the checklist, except in the discussion part; we did not include the limitations of the study related to incomplete retrieval of articles, as we did not face such a problem.

Supplementary 2 S2 file: Modified Newcastle-Ottawa Scale. This scale had been adapted from Wells et al. (2009), “The Newcastle-Ottawa Scale (NOS) for Assessing the Quality of Nonrandomized Studies in Meta-Analyses.” We considered the comparability to be controlled if the standard laboratory procedure is described in the articles. Since the outcome of the bacterial sensitivity test is obtained after full growth of the bacterial strains, we assigned one star for the assessment outcomes of all studies.
