# Supplementary material for: Drug Resistance Patterns of Escherichia coli in Ethiopia: A Meta-Analysis
Source: Biomed Res Int. 2018 May 6;2018:4536905. doi: 10.1155/2018/4536905 (PMC5960519; doi:10.1155/2018/4536905)
Supplement: Supplementary 1 — S1 file: PRISMA 2009 Checklist. We have conducted the meta-analysis according to Moher et al. (2009), “Preferred Reporting Items for Systematic Reviews and Meta-Analyses: The PRISMA Statement.” We included all the criteria on the checklist, except in the discussion part; we did not include the limitations of the study related to incomplete retrieval of articles, as we did not face such a problem. [file 4536905.f1.zip › 4536905.f1/3 A concise description of supplementary files_BMRI_2250673.docx]

**A concise description of supplementary files**

**S1 file:**

**PRISMA 2009 Checklist**

We have conducted the meta-analysis according to Moher *et al*., (2009), “Preferred Reporting Items for Systematic Reviews and Meta-Analyses: The PRISMA Statement”. We included all the criteria on the checklist, except in the discussion part, we did not include the limitations of the study related to incomplete retrieval of articles, as we did not face such a problem.

**S2 file:**

**Modified Newcastle Ottawa Scale**

This scale had adapted from Wells et al., (2009), “the Newcastle-Ottawa Scale (NOS) for assessing the quality of nonrandomized studies in meta-analyses”. We considered the comparability is controlled if the standard laboratory procedure is described in the articles. Since the outcome of the bacterial sensitivity test is obtained after full growth of the bacterial strains, we assigned one star for the assessment outcomes of all studies.
