# Supplementary material for: Drug Resistance Patterns of Escherichia coli in Ethiopia: A Meta-Analysis
Source: Biomed Res Int. 2018 May 6;2018:4536905. doi: 10.1155/2018/4536905 (PMC5960519; doi:10.1155/2018/4536905)
Supplement: Supplementary 2 — S2 file: Modified Newcastle-Ottawa Scale. This scale had been adapted from Wells et al. (2009), “The Newcastle-Ottawa Scale (NOS) for Assessing the Quality of Nonrandomized Studies in Meta-Analyses.” We considered the comparability to be controlled if the standard laboratory procedure is described in the articles. Since the outcome of the bacterial sensitivity test is obtained after full growth of the bacterial strains, we assigned one star for the assessment outcomes of all studies. [file 4536905.f2.zip › 4536905.f2/5 S2 file Newcastle Ottawa Scale E coli Drug resistancein Ethiopia_BMRI_2250675.pdf]

S2 file:

Modified Newcastle Ottawa Scale

Drug Resistance Patterns of *Escherichia coli* in Ethiopia: A meta-analysis

Kald Beshir Tuem[1*], Abadi Kahsu Gebre[1], Tesfay Mehari Atey[2], Helen Bitew[3], Ebrahim Mohammed Yimer[1], Derbew Fikadu Berhe[1]

Table Results of the critical appraisal of the included studies

Study (first author)	Study design	Selection				Comparability	Outcome		Quality Score out of 7
		Representativeness of the sample	Sample size	Ascertainment of exposure	Non-respondents	The subjects in different outcome groups are comparable, based on the study design or analysis. Confounding factors are controlled.	Assessment of outcome	Was follow-up long enough for outcomes to occur	
Abejew et al., 2014 [29]	RCS	1	1	1	1	0	1	1	6
Kibret et al., 2014 [30]	R	1	1	1	1	0	1	1	6
Kibret et al., 2011 [31]	R	1	1	1	1	0	1	1	6
Azene et al., 2011 [32]	R	1	1	1	1	0	1	1	6
Alebachew et al., 2016 [33]	CS	1	1	1	1	1	1	1	7
Alemu et al., 2012 [34]	CS	1	1	1	1	1	1	1	7
Wondimeneh et al., 2014 [35]	CS	1	1	1	1	0	1	1	6
Wondimu, 2013 [36]	CS	1	1	1	1	1	1	1	7
Eshetie et al., 2015 [37]	CS	0	0	1	1	0	1	1	4
Tiruneh et al., 2014 [38]	CS	0	0	1	1	0	1	1	4
Eshetie et al., 2016 [39]	CS	1	1	1	1	1	1	0	6
Mulu et al., 2017 [40]	R	1	1	1	1	0	1	1	6
Abera et al., 2014 [41]	CS	1	1	1	1	0	1	0	5
Adugna et al., 2015 [42]	CS	1	1	1	1	1	1	1	7
Demilie et al., 2012 [43]	CS	1	0	1	1	0	1	0	4
Derbie et al., 2017 [44]	R	0	1	1	1	0	1	1	5
Melaku et al., 2012 [45]	CS	1	1	1	1	0	1	1	6
Mulu et al., 2012 [46]	CS	1	1	1	1	1	1	0	6

Study (first author)	Study design	Selection				Comparability	Outcome		Quality Score out of 7
		Representativeness of the sample	Sample size	Ascertainment of exposure	Non-respondents	The subjects in different outcome groups are comparable, based on the study design or analysis. Confounding factors are controlled.	Assessment of outcome	Was follow-up long enough for outcomes to occur	
Mulu et al., 2015 [47]	CS	1	1	1	1	1	1	1	7
Dereje et al., 2017 [48]	CS	1	1	1	1	1	1	1	7
Dessie et al., 2016 [49]	CS	1	1	1	1	1	1	0	6
Desta et al., 2016 [50]	CS	1	1	1	1	1	1	1	7
Mamuye, 2016 [51]	CS	1	1	1	1	0	1	0	5
Legese et al., 2017 [52]	CS	1	1	1	1	1	1	0	6
Ramos et al., 2017 [53]	CS	1	1	1	1	0	1	0	5
Derese et al., 2016 [54]	CS	0	1	1	1	1	1	1	6
Beyene et al., 2011 [55]	CS	0	0	1	1	1	1	1	5
Debalke et al., 2014 [56]	CS	1	1	1	1	1	1	1	7
Mama et al., 2016 [57]	CS	1	1	1	1	0	1	1	6
Mulualem, 2012 [58]	CS	1	1	1	1	1	1	0	6
Zenebe et al., 2011 [59]	CS	1	1	1	1	0	1	1	6
Nigussie, 2017 [60]	CS	1	1	1	1	1	1	1	7
Amsalu et al., 2016 [61]	RSC	1	1	1	1	1	1	1	7
Tadesse et al., 2014 [62]	CS	1	1	1	1	1	1	0	6
Wasihun et al., 2015 [63]	PCS	0	1	1	1	1	1	1	6

This scale had adapted from Wells *et al.*, (2009), "the Newcastle-Ottawa Scale (NOS) for assessing the quality of nonrandomized studies in meta-analyses". We considered the comparability is controlled if the standard laboratory procedure is described in the articles. Since the outcome of the bacterial sensitivity test is obtained after full growth of the bacterial strains, we assigned one star for the assessment outcomes of all studies.
